# ﻿A new species of *Rhyncholagena* Lang, 1944 (Copepoda, Harpacticoida, Miraciidae) from Palau

**DOI:** 10.3897/zookeys.1180.109288

**Published:** 2023-09-20

**Authors:** Jisu Yeom, Wonchoel Lee

**Affiliations:** 1 Hanyang University, Department of Life Science, 222 Wangsimni-ro, Seoul 04763, Republic of Korea Hanyang University Seoul Republic of Korea; 2 Hanyang University, Research Institute for Natural Sciences, 222 Wangsimni-ro, Seoul 04763, Republic of Korea Hanyang University Seoul Republic of Korea

**Keywords:** 18S rRNA, Benthic copepod, Crustacea, meiofauna, mtCOI, taxonomy

## Abstract

A new species of Miraciidae Dana, 1846, *Rhyncholagenacuspis***sp. nov.**, was described from Palau. Morphological descriptions and gene fragment sequence barcoding were performed on the 11^th^ species of *Rhyncholagena* Lang, 1944 collected from sandy sediment samples in the subtidal zone of the Philippine Sea, Palau. Morphological characteristics were compared and an updated identification key was provided. A new species, *Rhyncholagenacuspis***sp. nov.**, was found to be morphologically similar to *Rhyncholagenalittoralis* Por, 1967 and *R.bermudensis* Malt, 1990. This is the first record of the genus *Rhyncholagena* in Palau. The study provides basic data for future studies and highlights the need for continued exploration of marine biodiversity in Palau and other regions.

## ﻿Introduction

Palau is renowned for its high biological diversity which is attributed to the influence of two currents: the North Equatorial Current and the North Equatorial Countercurrent passing through the area (Gopalakrishnan and Cornuelle 2019). Furthermore, coral reefs and marine biodiversity in Palau are well preserved (Friedlander et al. 2015). Several studies have been conducted on various marine organisms in Palau, such as coral reefs (Hamner et al. 2007), amphipods (Myers 2013, 2014), pontoniine shrimps (Marin and Paulay 2010), laomediid mud shrimps (Alvarez et al. 2010) and benthic dinoflagellates (Horiguchi et al. 2011). Moreover, studies on copepods such as calanoid copepods (Ohtsuka et al. 2000), misophrioid copepods (Boxshall and Iliffe 1990), siphonostomatoid copepods (Ho 1980), pelagic copepods in marine lakes (Saitoh et al. 2011) and symbiotic cyclopoid copepods (Kim and Boxshall 2020) have also been reported. However, there has been relatively little research on the marine harpacticoid copepods in the area.

In October 2018 and January 2019, meiofauna samples were collected from the subtidal zone of Palau via SCUBA diving and the benthic copepods inhabiting the Palau coast were identified. In the present study, we discovered a new species belonging to the genus *Rhyncholagena* in sandy sediments.

The genus *Rhyncholagena* Lang, 1944 belongs to the large family Miraciidae Dana, 1846, subfamily Diosaccinae Sars G.O., 1906, comprising 10 species and subspecies. The genus was established to accommodate three species, *R.lagenirostris* (Sars G.O., 1911), *R.spinifer* (Farran, 1913) and *R.pestai* (Monard, 1935), previously assigned to *Amphiascus* Sars G.O., 1905. *Rhyncholagena* is distinguished from *Amphiascus* by subtle morphological characteristics, such as the flask shape of the rostrum (Lang 1948). *Rhyncholagena* is characterised by a bottle-like or elongated rostrum and an incision between the apical setae of the fifth pereiopod exopod (Por 1967).

In this study, we discovered a new species of the genus *Rhyncholagena* for the first time in Palau. It is the 11^th^ member of *Rhyncholagena* and morphologically similar to *R.littoralis* Por, 1967 and *R.bermudensis* Malt, 1990. The most prominent feature of the new species is the development of a long lateral spinous process at the end of the anal somite. Herein, we provide taxonomic description of the new species and a revised identification key to the *Rhyncholagena* species. Additionally, we obtained the 18S ribosomal RNA (18S rRNA) and mitochondrial cytochrome oxidase I (mtCOI) sequences from the new species.

## ﻿Material and methods

**Figure 1.**
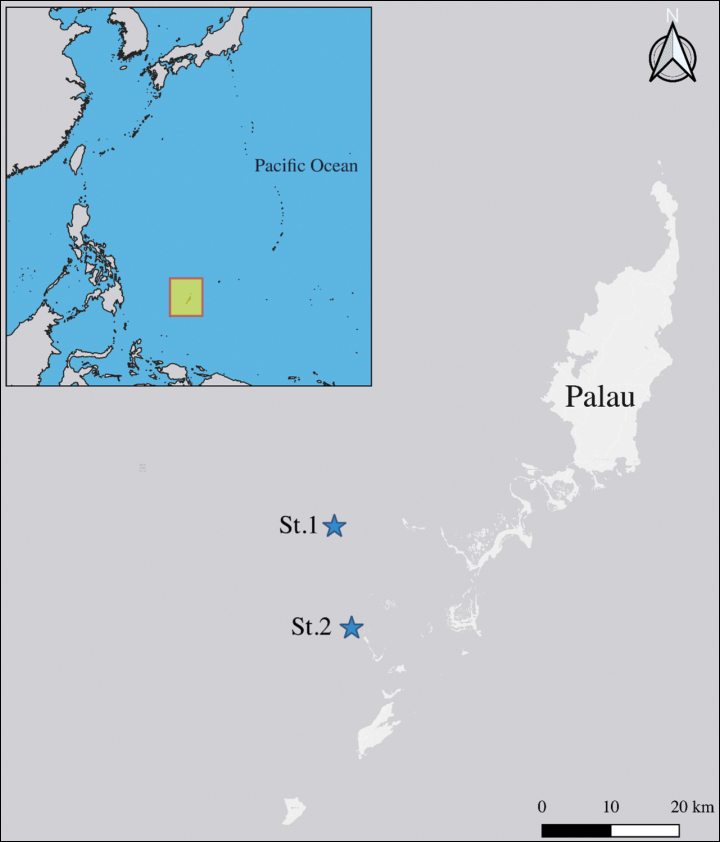
Map of type localities.

**Figure 2.**
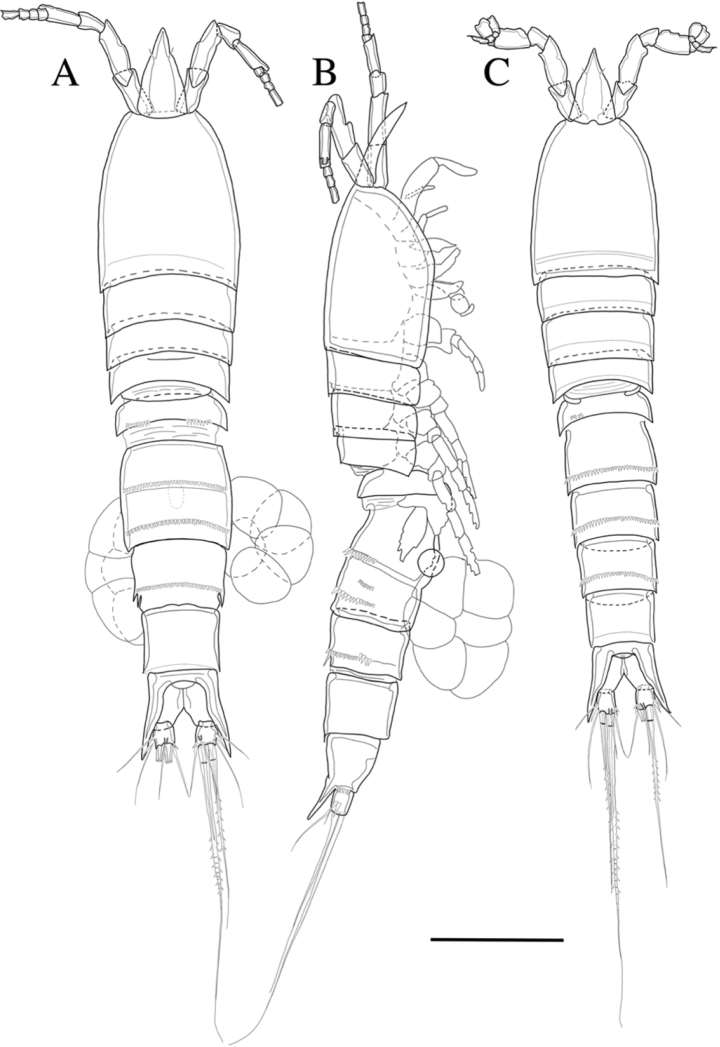
*Rhyncholagenacuspis* sp. nov. Female **A** habitus, dorsal **B** habitus, lateral. Male **C** habitus, dorsal. Scale bar: 100 μm.

**Figure 3.**
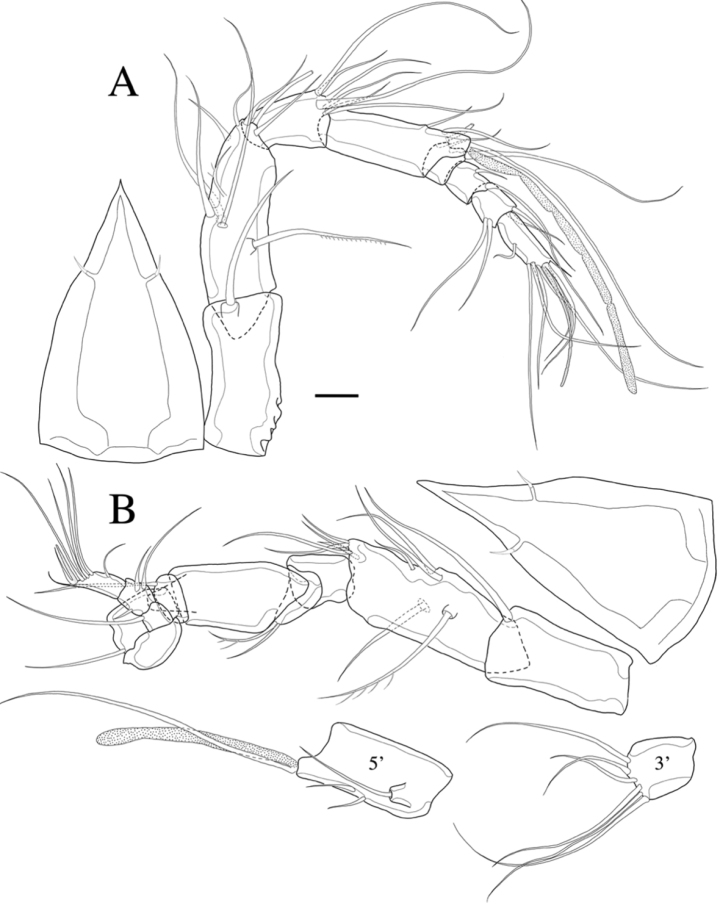
*Rhyncholagenacuspis* sp. nov. Female **A** A1 and rostrum, dorsal. Male **B** A1 and segments (3^rd^ and 5^th^ segment), rostrum, dorsal. Scale bar: 10 μm.

**Figure 4.**
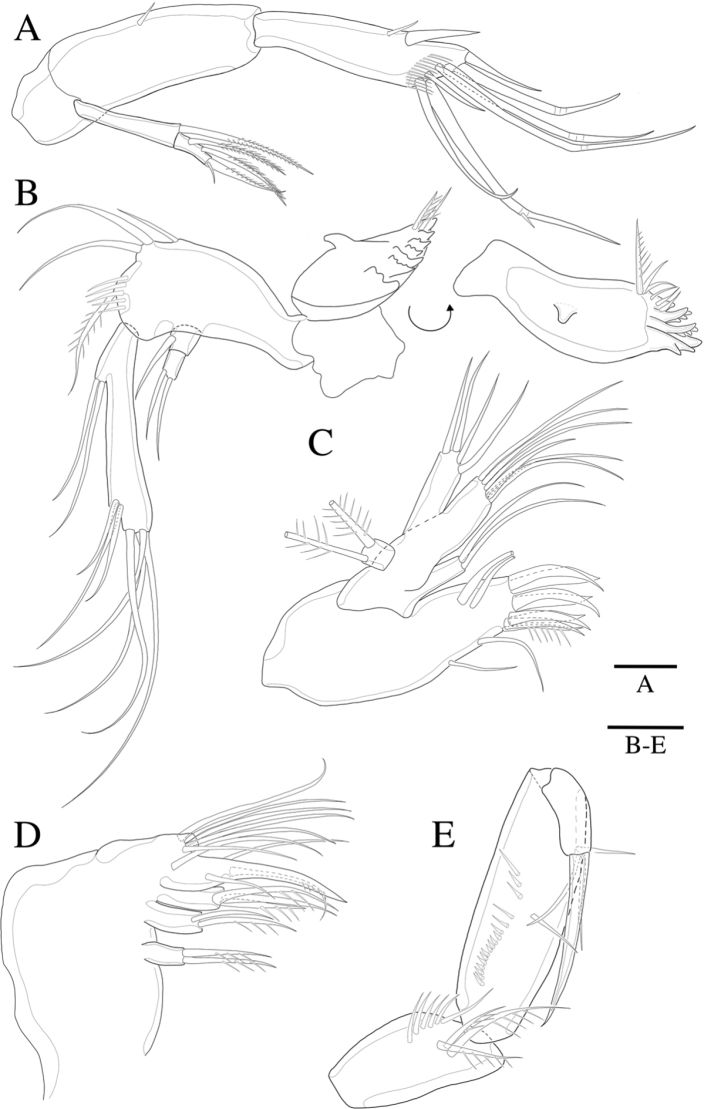
*Rhyncholagenacuspis* sp. nov. **A** A2 **B** mandible **C** maxillule **D** maxilla **E** maxilliped. Scale bars: 10 μm.

**Figure 5.**
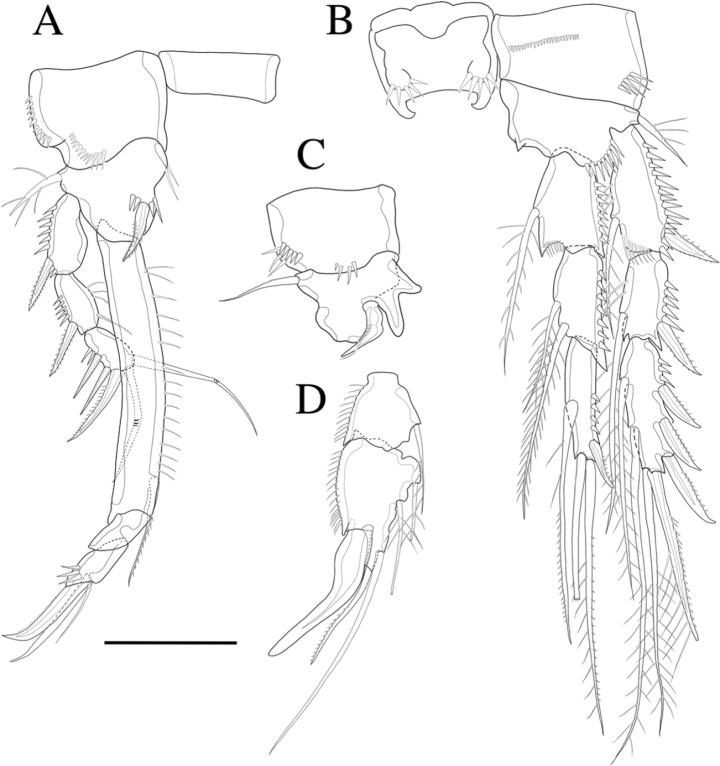
*Rhyncholagenacuspis* sp. nov. Female **A** P1 **B** P2. Male **C** P1 basis **D** P2 endopod. Scale bar: 30 μm.

**Figure 6.**
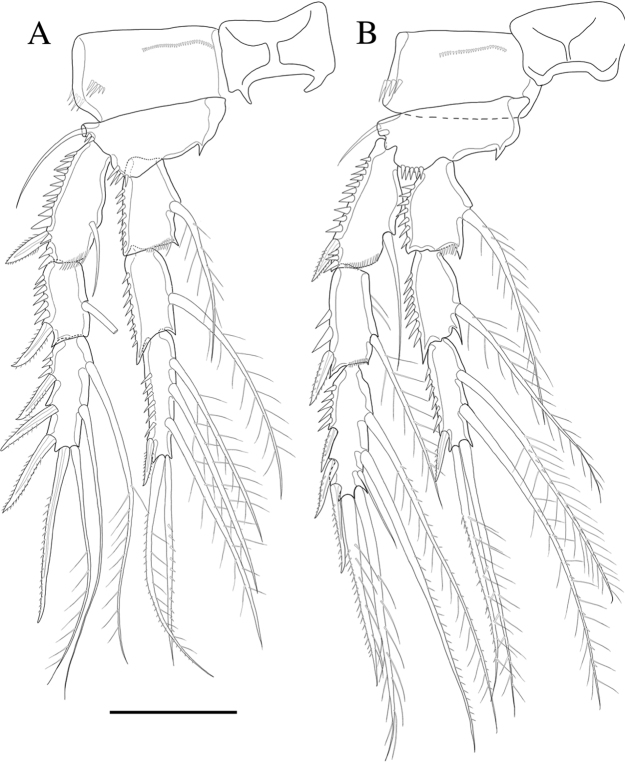
*Rhyncholagenacuspis* sp. nov. **A** P3 **B** P4. Scale bar: 30 μm.

**Figure 7.**
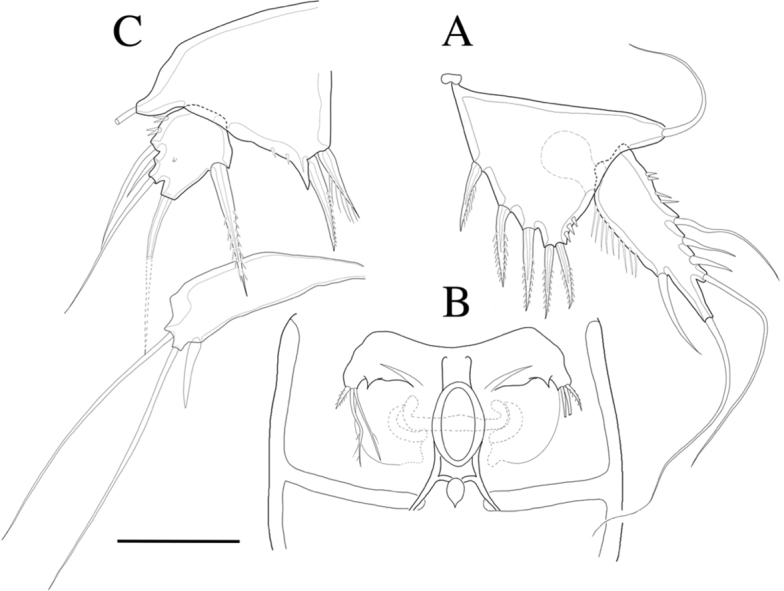
*Rhyncholagenacuspis* sp. nov. Female **A** P5 **B** genital field and P6. Male **C** P5 and P6. Scale bar: 30 μm.

Sediment samples were obtained from two stations along the west coast of Palau (Fig. 1) at a depth of 15 m using SCUBA diving techniques during October 2018 and January 2019. The sandy sediments were then rinsed with fresh water and the supernatant was filtered through a 38 μm sieve before being fixed with 99% ethanol. Harpacticoid specimens were sorted under a dissecting microscope (Olympus SZX12) and stored in 99% ethanol at 4 °C. The identification of harpacticoids was carried out by following Huys et al. (1996) and Wells (2007) using a compound microscope (Olympus BX51) at 400–1000× magnification. All drawings (Figs 2–7) were prepared by using a drawing tube on an Olympus BX51 differential interference contrast microscope.

**Figure 8.**
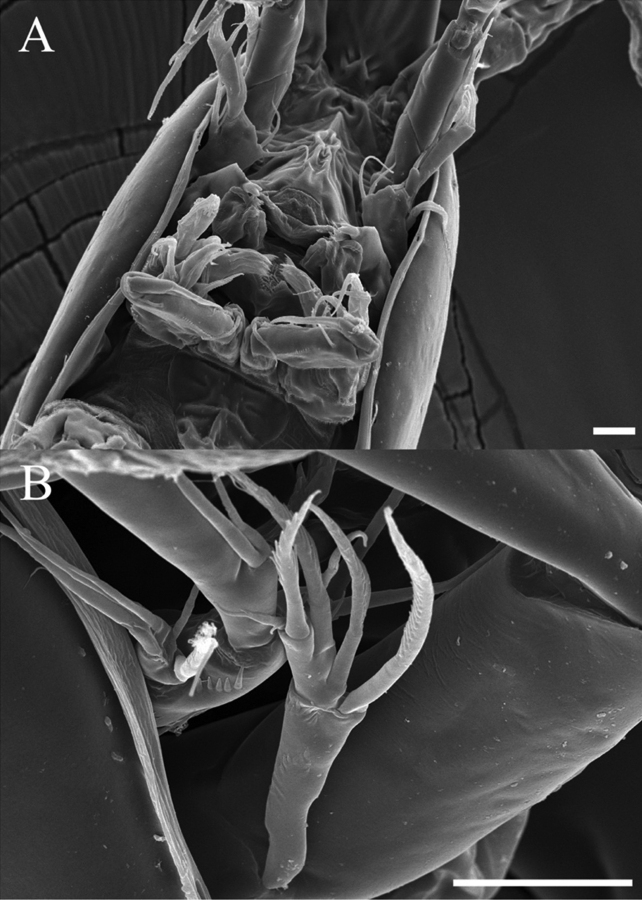
*Rhyncholagenacuspis* sp. nov. SEM photographs **A** mouth parts **B** A2 exopod. Scale bars: 10 μm.

**Figure 9.**
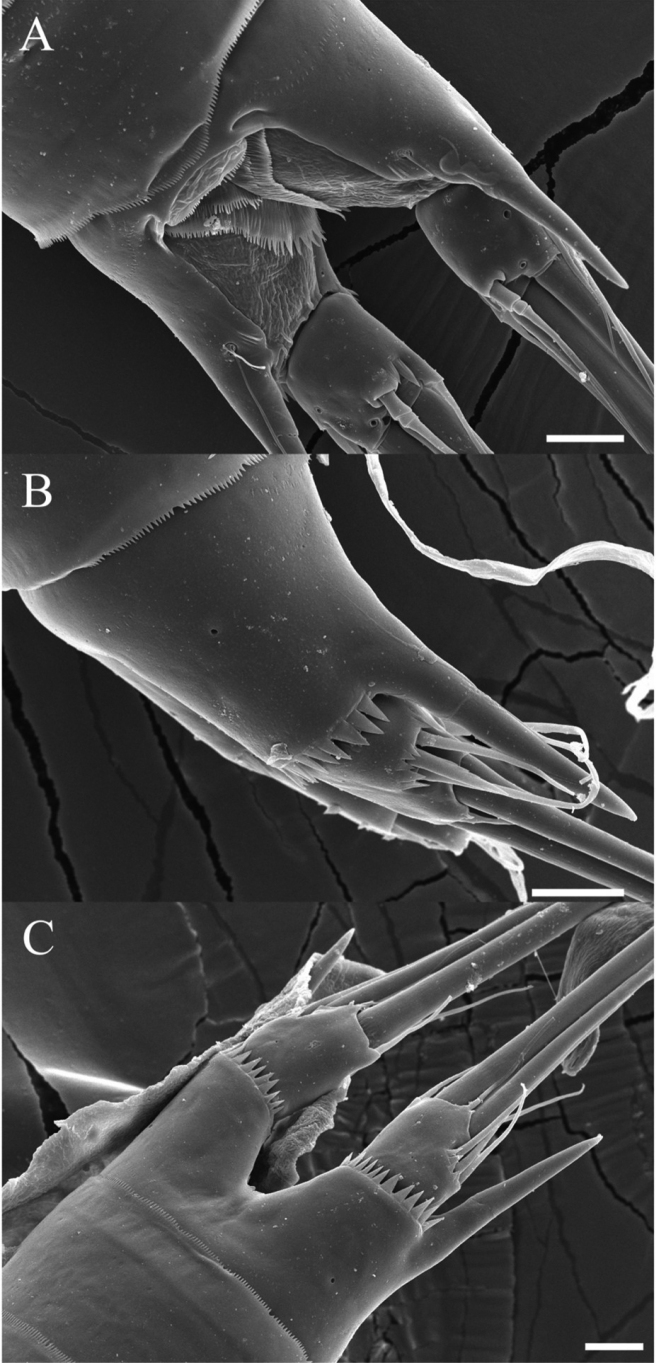
*Rhyncholagenacuspis* sp. nov. SEM photographs **A** anal somite and caudal rami, dorsal **B** caudal ramus, lateral **C** anal somite and caudal rami, ventral. Scale bars: 10 μm.

**Figure 10.**
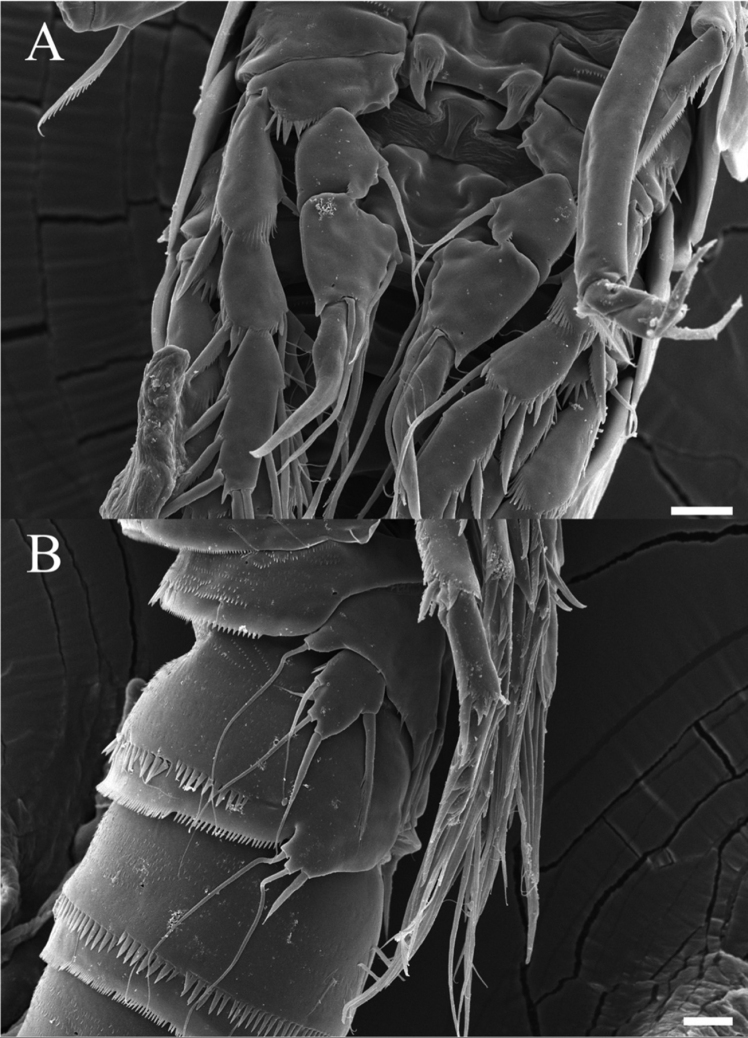
*Rhyncholagenacuspis* sp. nov. SEM photographs. Male **A** P2 **B** P5 and P6. Scale bars: 10 μm.

Scanning electron micrographs (Figs 8–10) were taken with the Hitachi S-3400N scanning electron microscope (SEM). Specimens were prepared for SEM by being transferred into pure isoamyl-acetate, then critical-point dried, mounted on stubs, coated in gold and observed under SEM on the in-lens detector at an accelerating voltage of 10.0 kV and 15.0 kV and working distance between 7.0 to 13.4 mm. Digital photographs were processed and combined into plates using Adobe Photoshop CS6.

The descriptive terminology follows Huys et al. (1996). Abbreviations used in the text are as follows: exp, exopod; enp, endopod; P1–P6, first to sixth thoracic legs (thoracopods); exp (enp)-1 (2, 3) to denote the proximal (middle, distal) segment of a ramus. Specimens were deposited in the collection of the National Marine Biodiversity Institute of Korea (MABIK), voucher codes: MABIKCR00254070–MABIKCR00254077.

To extract DNA templates, we used worm lysis buffer as described in Williams et al. (1992). We then amplified fragments from two genes, 18S rRNA and mtCOI genes, using polymerase chain reaction (PCR) premix (BIONEER Co. / Labopass, Korea) and 3 μl of genomic DNA as a template. For mtCOI, we used the primers, Cop-CO1-2189R (Bucklin et al. 2010) and LCO 1490 (Folmer et al. 1994), while for 18S rRNA, we used 18S-F1, 18S-F3, 18S-R7 and 18S-R9 (Yamaguchi and Endo 2003). We followed the protocols specified in the references for PCR amplification. Successful amplification was confirmed by 1% agarose gel electrophoresis. PCR products were sent to Macrogen (Seoul, Korea) for purification and DNA sequencing. DNA was sequenced on an ABI automatic capillary sequencer using the same sets of primers as those used for amplification.

**Figure 11.**
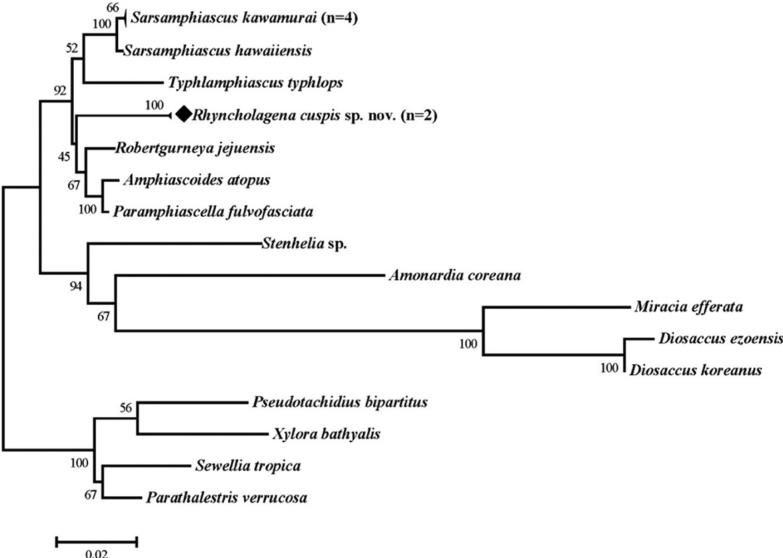
Maximum Likelihood phylogenetic tree including ten genera of Miraciidae and outgroups, based on 18S rRNA sequences. Tree shows bootstrap values (%). (n: the number of sequences contained).

The phylogenetic tree (Fig. 11) was constructed using the Maximum Likelihood method. The analysis of 18S rRNA involved 20 nucleotide sequences. Sequences of the Miraciidae and the outgroups were obtained from NCBI (Table 1). The obtained sequences were checked manually and aligned using the ClustalW algorithm (Thompson et al. 1994) in MEGA version 7.0 (Kumar et al. 2016). Maximum Likelihood analysis was performed using the Kimura 2-parameter model and Gamma distributed with Invariant sites (K2+G+I) (Kimura 1980; Nei and Kumar 2000), based on the model test results in MEGA. One thousand bootstrap replicates were performed to obtain a relative measure of node support for the resulting trees.

**Table 1.** GenBank numbers of 18S rRNA sequences used in phylogenetic analyses in this study.

Family	Genus, Species	Author, Year	NCBI number
Miraciidae Dana, 1846	*Amphiascoidesatopus*	Lotufo & Fleeger, 1995	KC815328
	*Paramphiascellafulvofasciata*	Rosenfield & Coull, 1974	EU380293
	*Typhlamphiascustyphlops*	(Sars G.O., 1906)	EU380292
	*Sarsamphiascuskawamurai*	(Ueda & Nagai, 2005)	MN541391–MN541394
	*Sarsamphiascushawaiiensis*	Yeom & Lee, 2020	MN496456
	*Stenhelia* sp.		EU380291
	*Amonardiacoreana*	Song, Rho & Kim W., 2007	KT030261
	*Diosaccusezoensis*	Itô, 1974	KR048740
	*Diosaccuskoreanus*	Lim, Bang, Moon & Back, 2020	MT002900
	*Miraciaefferata*	Dana, 1849	EU380294
	*Robertgurneyajejuensis*	Yeom & Lee, 2022	OP798781
	*Rhyncholagenacuspis* sp. nov.	Yeom & Lee, 2023	OR257802–OR257803
Dactylopusiidae Lang, 1936	*Sewelliatropica*	(Sewell, 1940)	EU380299
Thalestridae Sars G.O., 1905	*Parathalestrisverrucosa*	Itô, 1970	MT002906
Pseudotachidiidae Lang, 1936	*Pseudotachidiusbipartitus*	Montagna, 1980	MF077760
	*Xylorabathyalis*	Hicks, 1988	MF077748

## ﻿Results

### ﻿Systematics

**Class Copepoda H. Milne-Edwards, 1840**

**Order Harpacticoida Sars G.O., 1903**

**Family Miraciidae Dana, 1846**

**Subfamily Diosaccinae Sars G.O., 1906**

**Genus *Rhyncholagena* Lang, 1944**

#### ﻿ Rhyncholagena cuspis sp. nov.

Taxon classificationAnimaliaHarpacticoidaMiraciidae

C603497E-CEE8-568C-9189-88F27C18305E

https://zoobank.org/F595B4B3-DA93-418F-99E8-3613B31ECDD1

Figs 2 , 3 , 4 , 5 , 6 , 7 , 8 , 9 , 10

##### Type locality.

St.1-Shark City (7°16'04.2"N, 134°11'11.4"E) and St.2-Blue Corner (7°08'04.0"N, 134°13'13.5"E) diving sites in west coast of Palau on 19–20 October 2018 and 22–26 January 2019, collected by Wonchoel Lee, Eunha Choi and Jisu Yeom. Sediment type: sand. Depth: 15 m.

##### Type material.

***Holotype***: Palau • ♀; Koror, Shark City diving site; 7°16'04.2"N, 134°11'11.4"E, depth 15 m, 20 Oct 2018; W. Lee leg.; SCUBA diving; GenBank: OR252612, OR257802; MABIKCR00254070. ***Allotype***: Palau • ♂; same collection data as for holotype; GenBank: OR252613, OR257803; MABIKCR00254071. ***Paratypes***: Palau • ♀; Peleliu, Blue Corner diving site; 7°08'04.0"N, 134°13'13.5"E, depth 15 m, 22 Jan 2019; W. Lee, E. Choi, J. Yeom leg.; SCUBA diving; MABIKCR00254072 • ♀,♂; Peleliu, Blue Corner diving site; 7°08'04.0"N, 134°13'13.5"E, depth 15 m, 19 Oct 2018; W. Lee leg.; SCUBA diving; MABIKCR00254073, MABIKCR00254074 • ♀,♂; Koror, Shark City diving site; 7°16'04.2"N, 134°11'11.4"E, depth 15 m, 26 Jan 2019; W. Lee, E. Choi, J. Yeom leg.; SCUBA diving; MABIKCR00254075, MABIKCR00254076 • 2♀,5♂; same collection data as for holotype; MABIKCR00254077.

##### Description.

**Female.** Total body length 593 µm (n = 4) (Fig. 2A); body slender, cylindrical, slightly tapering distally. Rostrum (Figs 2A, B, 3A) prominent, triangular flask-shaped, tapering distally, reaching second antennular segment, defined at base; with two small sensilla. Cephalothorax bell-shaped (Fig. 8A). Anal somite (Figs 2, 9) with row of spinules along ventral distal margin and long lateral spinous process distally, 1.5 times longer than caudal ramus; Anal operculum well developed, semicircular.

Caudal ramus (Figs 2A, B, 9). Approximately 1.2 times as long as greatest width, armed with spinules on the lateral ventral surface and the inner dorsal surface. Each ramus armed with seven setae; seta I and seta II located the medial of the lateral margin, seta I bare, seta II short, seta III bare and located near seta I and seta II on the lateral margin; seta IV well developed, bipinnate, seta IV more than half as long as seta V, seta V as long as urosome; both terminal caudal setae with fractured plane; seta VI bare, located on inner distal corner; seta VII, bare, located on dorsal surface of caudal ramus.

A1 (Fig. 3A). Eight-segmented, slender; seg-2 about 2.5 times as long as broad and slightly longer than seg-1; seg-4 twice as long as seg-3. Armature formula (segment-[number of seta/setae]): 1-[1 bare], 2-[8 bare + 2 pinnate], 3-[7 bare], 4-[4 bare + aesthetasc], 5-[2 bare], 6-[2 bare], 7-[4 bare], 8-[5 bare + acrothek]. Aesthetasc on seg-4 fused basally with adjacent seta and about twice as long as distal four segments combined. Apical acrothek consisting of aesthetasc and two bare setae.

A2 (Fig. 4A). Basis short, squarish, unarmed. Endopod two-segmented, enp-2 with two spiniform setae laterally, four geniculate setae and three bare setae terminally. Exopod (Fig. 8B) two-segmented, exp-1 with the long pinnate seta; exp-2 with two pinnate setae laterally, pinnate seta and small bare seta distally.

Mandible (Fig. 4B). Gnathobase bearing chitinous projection on surface; basis with setules bearing three bare setae and one pinnate seta; exopod two-segmented, exp-1 with seta, exp-2 with two setae; endopod bearing two setae on proximal lobe and seven terminal setae.

Maxillule (Fig. 4C). Praecoxal arthrite bearing six elements distally with two pinnate spines, two bare setae laterally and two bare setae on surface; coxa with two bare setae; basis with six bare setae; endopod bearing four bare setae; exopod bearing two plumose setae.

Maxilla (Fig. 4D). Three endites of syncoxa with two, two and three setae, proximal to distal, respectively; allobasis transformed to thick pinnate claw bearing bare seta, two thin setae and short seta on surface; uniarticulated endopod with six bare setae.

Maxilliped (Fig. 4E). Subchelate; syncoxa with three pinnate setae and row of setules; basis with row of setules on surface, three times as long as broad, bearing two bare setae; endopod elongate, with strong claw and two bare setae.

In P1–P4, all rami three-segmented and coxa ornamented with several rows of spinules. Armature formula of the new species as follows:

P1 (Fig. 5A). Basis with outer ornamented seta, inner margin with setules and armed with bipinnate spine. Exp-1 inner margin bare, exp-1 and exp-2 with bipinnate outer spine and ornamented with spinules along outer margin, exp-2 inner margin with setules and bare inner seta; exp-3 with three outer spines and two geniculate setae distally. Enp-1 much longer than exopod, about 6.2 times longer than wide, with inner pinnate seta distally, which almost reaches the end of enp-3, inner margin ornamented with setules; enp-2 small and bare; enp-3 as long as enp-2, outer margin ornamented with spinules, bearing slender bare seta at inner distal edge, long seta and unipinnate claw-like spine apically.

P2 (Fig. 5B). Basis with setules distally and outer ornamented seta. Both exp-1 and exp-2 with inner plumose seta, inner margin ornamented with setules, bipinnate outer spine and outer margin ornamented with spinules; exp-3 with two plumose inner setae, long plumose seta at inner terminal, long spiniform seta with plumose inner side and pinnate outer side at outer terminal and three outer spines, proximal outer margin ornamented with spinules. Endopod as long as exopod, enp-1 with plumose inner seta; enp-2 with two plumose inner setae; enp-3 with pinnate inner seta, two setae distally and spine at outer distal corner, outer margin of endopod segments ornamented with spinules.

P3 (Fig. 6A). Basis with bare outer seta. Both exp-1 and exp-2 with inner seta and outer margin ornamented with spinules; exp-3 with two long inner setae, long plumose seta and long pinnate seta at inner terminal and three outer pinnate spines, proximal outer margin ornamented with spinules. Endopod as long as exopod, both enp-1 and enp-2 with plumose inner seta; enp-3 with three plumose inner setae, two setae distally and short spine at outer distal corner; outer margin of endopod segments ornamented with spinules.

P4 (Fig. 6B). Basis with bare outer seta. Exp-1 with bare inner seta; exp-2 with plumose inner seta, inner margin ornamented with setules; exp-3 with two long setae and short seta on inner margin, long plumose seta and long seta with plumose inner side and pinnate outer side at distal and three outer spines, outer margin of exopod ornamented with spinules. Endopod shorter than exopod, both enp-1 and enp-2 with plumose inner seta; enp-3 with two plumose inner setae, two setae distally and spine at outer distal corner, outer margin of endopod segments ornamented with spinules.

P5 (Fig. 7A). Baseoendopod and exopod distinct, baseoendopod with long, slender and bare basal seta; endopodal lobe bearing three inner pinnate spines and two distal pinnate spines. Exopod elongated, 3.6 times longer than wide, with incision between the apical setae, with setules along inner margin and spinules along outer margins, bearing six bare setae.

Genital area as in Fig. 7B. P6 with two long setae and short pinnate spiniform seta. Genital double-somite with epicopulatory bulb.

**Male.** Body (Fig. 2C) 502 µm (n = 3) in length, smaller and more slender than female. Sexual dimorphism shown in A1, P1, P2, P5 and P6.

A1 (Fig. 3B). Subchirocer, 11-segmented. Armature formula: 1-[1 bare], 2-[9 bare + 1 pinnate], 3-[6 bare], 4-[1 bare + 1 pinnate], 5-[4 bare + aesthetasc], 6-[1 bare], 7-[1 bare], 8-[0], 9-[1 bare], 10-[3 bare], 11-[4+1 acrothek].

P1 basis (Fig. 5C) with claw-like spine and inner margin with bifid protrusion.

P2 endopod (Figs 5D, 10A). Two-segmented, outer margin ornamented with setules; enp-1 with pinnate inner seta; enp-2 modified, bearing three inner setae, terminal seta, outer distal curved thick spine.

P5 (Figs 7C, 10B). Baseoendopod and exp distinct; endopodal lobe ornamented with spinules at outer margin, armed with two spines. Exp with five setae in total, including bipinnate inner seta, long distal bare seta and three bare outer setae, outer margin ornamented with setules.

P6 (Figs 7C, 10B) represented by short inner seta and two long bare setae on outer distal corner of genital operculum.

##### Etymology.

The scientific name is derived from the Latin *cuspis* (meaning a point), alluding to the spinous process on the anal somite.

##### DNA-barcode.

The 18S rRNA sequences [female: 1,691 base pairs (bp) (OR257802); male: 1,775 bp (OR257803)] and the mtCOI sequences [female: 641 bp (OR252612); male: 573 bp (OR252613)] were successfully amplified from the two specimens of the new species. These sequences were submitted to GenBank.

### ﻿Identification key

Ma and Li (2018) suggested a key for *Robertgurneya* species, based on earlier keys provided by Lang (1948) and Wells (2007). The format of the existing identification key was maintained and minor modifications and additions to the new species were reflected in this study. The updated identification key below includes 11 species and subspecies of *Rhyncholagena*. All valid species of the genus are listed in Table 2 and their morphological characters are being compared.

**Table 2.** Morphological comparison of species within *Rhyncholagena* (*not exceeding half-length of exp: X / exceeding half-length of exp: O / slightly exceeding half-length of exp: ∆).

Species	Rostrum	A2 exp seg: setae	Setal formulae of swimming legs (exp/enp)						♀ P5	
			P1	P2	P3	P4	P5 (♀)	P5 (♂)	Exp long/ width	Distal of benp*
***R.bermudensis* Malt, 1990**	“Reaching level of third segment of A1”	2:3	0.1.122/ 1.1.021	1.1.223/ 1.2.130	1.1.223 (2)/ 1.1.231	1.1.323/ 1.1.230	6/5	–	≈3	X
***R.josaphatis* Por, 1967**	“Peculiar shape”; reaching level of second segment of A1	3:6	0.1.122/ 1.0.120	1.1.223/ 1.2.121	1.1.223/ 1.2.321	1.1.323/ 1.1.221	6/4	5/2	≈1.9	O
***R.lagenirostris* (Sars, 1911)**	flask shape; reaching mid-level of second segment of A1	3:5	0.1.122/ 1.1.120	1.1.123/ 1.2.121	1.1.123/ 1.1.321	1.1.223/ 1.1.221	6/5	5/3	≈2.4	△
***R.levantina* Por, 1964**	“Strongly convex and its tip is only slightly produced”; “biconvex; apical portion terminating in a very fine point” (Wells 2007); reaching mid-level of second segment of A1	-:-	0.1.122/ 1.1.120	1.1.123/ 1.1.121	1.1.123/ 1.1.321	1.1.323/ 1.1.221	5/5	5/2	≈2.2	O
***R.littoralis* Por, 1967**	“Narrowly trianglar and very pointed”; reaching level of fourth segment of A1	2:4	0.1.122/ 1.0.120	1.1.223/ 1.2.121	1.1.223/ 1.1.321	1.1.323/ 1.1.221	6/5	–	≈1.6	X
***R.pestaipestai* (Monard, 1935)**	“Flask-shaped; bulbous proximally then tapering to an extremely fine point (Wells 2007)”; “apical portion very narrow, terminating in a very fine point *Rhyncholagenapestai* [sensu Por 1964]” (Wells 2007)	3:-	0.1.122/ 1.1.120	1.1.223/ 1.2.121	1.1.223/ 1.2.321	1.1.323/ 1.1.221	6/5	6/3	≈2.1	△
***R.pestaiamericana* Rouch, 1962**	“Bottle-shaped”; “flask-shaped; bulbous proximally then tapering to an extremely fine point (Wells 2007)”	3:-	0.1.122/ 1.1.120	1.1.223/ 1.2.121	1.1.223/ 1.1.321	1.1.323/ 1.1.221	6/5	–	≈2.5	O
***R.profundorum* Por, 1967**	“Fairly produced”; “approximately triangular; apex extremely finely pointed” (Wells 2007); reaching level of second segment of A1	3: 5–6	0.1.122/ 1.1.120	1.1.223/ 1.2.121	1.1.223/ 1.2.321	1.1.323/ 1.1.221	5/5	–	≈1.9	X
***R.spinifer* (Farran, 1913)**	reaching level of second segment of A1	3:–	0.1.122/ 1.0.120	1.1.123/ 1.2.121	1.1.123/ 1.1.321	1.1.223/ 1.1.221	6/5	5/3	≈2.9	△
***R.paraspinifer* Ma & Li, 2018**	“Almost triangular”; reaching level of second segment of A1	2:4	0.1.122/ 1.1.120	1.1.223/ 1.2.121	1.1.223/ 1.1.321	1.1.323/ 1.1.221	5/5	4/2	≈1.2	X
***R.cuspis* sp. nov.**	flask shape; reaching level of second segment of A1	2:5	0.1.122/ 1.0.120	1.1.223/ 1.2.121	1.1.223/ 1.1.321	1.1.323/ 1.1.221	6/5	5/2	≈3	X

### ﻿Key to the species of the *Rhyncholagena*

**Table:** 

1	P5 baseoendopod with four setae	***R.josaphatis* Por, 1967**
–	P5 baseoendopod with five setae	**2**
2	P5 exopod with five setae	**3**
–	P5 exopod with six setae	**5**
3	P3 enp-2 with two inner setae	***R.profundorum* Por, 1967**
–	P3 enp-2 with one inner seta	**4**
4	P2 enp-2 with two inner setae	***R.paraspinifer* Ma & Li, 2018**
–	P2 enp-2 with one inner seta	***R.levantina* Por, 1964**
5	P2–P3 exp-3 with one inner seta	**6**
–	P2–P3 exp-3 with two inner setae	**7**
6	The second segment of A1 produced a well-marked and incurved spinous projection in middle inside; P5 exp more than twice as long as wide	***R.lagenirostris* (Sars G.O., 1911)**
–	The second segment of A1 without spinous projection in middle inside; P5 exp about 3 times as long as wide	***R.spinifer* (Farran, 1913)**
7	P3 enp-2 with two inner setae	***R.pestaipestai* (Monard, 1935)**
–	P3 enp-2 with one inner seta	**8**
8	P5 exopod with two apical projections, longer one about two times as long as shorter one	**9**
–	Two apical projections of P5 exopod mostly as long as each other	**10**
9	A2 exopod with three setae; P1 enp-2 with seta	***R.bermudensis* Malt, 1990**
–	A2 exopod with five setae; P1 enp-2 without seta; long lateral spinous process at the end of the anal somite	***Rhyncholagenacuspis* sp. nov.**
10	P5 exopod less than two times as long as greatest wide; the length of caudal rami shorter than width	***R.littoralis* Por, 1967**
–	P5 exopod more than two times as long as greatest wide; the length of caudal rami longer than width	***R.pestaiamericana* Rouch, 1962**

## ﻿Discussion

### ﻿Morphology

The new species can be placed in the genus *Rhyncholagena*, based on two characteristics: the incision between the apical setae of the P5 exp and the elongated rostrum. A notable trait that appears only in the new species within the genus is a spinous process on the anal somite. This character is suggested as autapomorphy of the new species.

Based on the discussion presented by Ma and Li (2018), we assigned the new species to group 1 as it lacks a seta in the P1 enp-2. However, according to the identification key provided in the same paper, the new species was identified as *R.bermudensis* belonging to group 2. The morphological differences between these species are as follows: (1) The long lateral spinous process at the end of the anal somite (absent in *R.bermudensis*), (2) The length of the rostrum (reaching the level of the third segment of A1 in *R.bermudensis*, but only the middle of the second segment in the new species), (3) The number of setae on the A2 exopod (two more in the new species), (4) P1 enp-2 seta (long in *R.bermudensis*).

Following the keys to harpacticoid species (Wells 2007), this copepod was identified as *R.littoralis* by the following two characteristics: A2 exp with two segments and setal formulae of swimming legs. However, they were distinguished by a combination of the following morphological characteristics: (1) The long lateral spinous process at the end of the anal somite (absent in *R.littoralis*), (2) The type of apical setae on the female P5 benp (plumose in the new species), (3) The length-width ratio of the female P5 exp (elongated in the new species), (4) The number of setae on the mandibular palp (more setae in the new species), (5) The segmentation of the exopod of the mandible (more segments in the new species), (6) The number of setae on basis and endopod of the maxillule (three more in the new species) and (7) The number of endites in the maxilla (four endites, one more in *R.littoralis*).

The new species tends to have more setae or segments in the mouthparts and more ornamentations than *R.littoralis*. It supports the possibility that the new species may be more ancestral than *R.littoralis* in terms of oligomerization. In addition, the factors of morphological differences in relation to the habitat can be considered. It has been reported to be distributed in gravel bottoms in shallow waters of the Red Sea (Por 1967) and the coral reef area of the Atlantic Ocean (Sarmento and Santos 2012). It can be inferred that, as the habitat changed from the subtidal zone with strong currents to shallow areas with weak currents, the general evolutionary trend of crustaceans may have occurred, such as the reduction of the setae and decorations of copepods and fusion of segments. It is assumed that the major factor in the change in the morphology of the mouthparts is the influence of changes in the feeding process. *R.littoralis*, which is known to inhabit areas with more gravel than the sandy environments where the new species inhabits, may filter less during its feeding process and, thus, it would have been less necessary to maintain abundant setae on its mouthparts in these environments.

### ﻿Distribution

Considering the distribution records of the previously reported 10 species (Ma and Li 2018), this genus does not have any specificities for a specific regional distribution (Atlantic Ocean, Indian Ocean, Indo-Pacific Region, Red Sea and Mediterranean Sea), specific environmental distribution (mangrove, coral reefs and seagrass), depth (intertidal to subtidal zone, 0.5–700 m) and particle size of sediment (mud, sand and gravel).

Considering these distribution records, this genus has the potential to thrive in diverse regions and environments. The low specificity of region and habitat environment and high ecological flexibility suggest the possibility that this taxon can adapt well to environmental changes. Further research on this taxon through collection and excavation studies will not only allow us to find the forms of the genus that appear universally in various environments, but also provide evidence of evolutionary trends within taxa depending on the habitat.

### ﻿Phylogeny

To infer the phylogenetic position of the genus *Rhyncholagena* within Miraciidae, a phylogenetic tree (Fig. 11) was constructed using the 18S rRNA sequences of Miraciidae species uploaded to the NCBI database. According to the phylogenetic tree, this genus is relatively close to genera, such as *Amphiascoides* Nicholls, 1941, *Paramphiascella* Lang, 1944 and *Robertgurneya* Apostolov & Marinov, 1988, within the subfamily Diosaccinae. The low bootstrap value of this clade implies that there are still numerous gaps in our understanding of the systematics, as the genera included within the phylogenetic tree are of limited diversity. Nevertheless, *Rhyncholagena* is relatively distant from *Diosaccus* Boeck, 1873 and *Amonardia* Lang, 1944. This is consistent with the overall trend of the phylogenetic tree, which was created by Lang (1944), based on morphological characteristics. From a morphological standpoint, *Diosaccus* and *Amonardia* belong to a different clade from the other six genera in Diosaccinae included in the phylogenetic tree (Fig. 11). Their morphological differences include more inner setae in P3 exp-3 (three setae) and fewer setae in the P5 exp of males (four setae).

Further research is needed for detailed phylogenetic considerations; however, because the two genera, *Diosaccus* and *Amonardia*, were confirmed to be more closely related to subfamilies other than Diosaccinae, it is judged that the phylogenetic re-establishment of the family Miraciidae is necessary.

As a result of calculating the p-distance of the sequences obtained in this study and the mtCOI sequences of Miraciidae species uploaded to NCBI, it was confirmed that the sequences of the new species differed by more than 20% from those of the other species. Highlighting the novelty of this study, the genetic information of *Rhyncholagena* was registered in NCBI for the first time. These data will not only serve as a future basis for phylogenetic studies of the family Miraciidae, but also for the benthic environmental biodiversity of Palau.

## Supplementary Material

XML Treatment for Rhyncholagena cuspis

## ﻿Additional information

### Author ORCIDs

Jisu Yeom https://orcid.org/0000-0002-5651-6036

Wonchoel Lee https://orcid.org/0000-0002-9873-1033

### Data availability

All of the data that support the findings of this study are available in the main text.

## References

[B1] Alvarez F Villalobos JL Iliffe TM (2010) A new species of mud shrimp of the genus *Espeleonaushonia* Juarrero & Martínez-Iglesias, 1997 (Decapoda: Gebiidea: Laomediidae) from Palau. Zootaxa 2429(1): 61–68. 10.11646/zootaxa.2429.1.5

[B2] Boxshall GA Iliffe TM (1990) Three new species of misophrioid copepods from oceanic islands. Journal of Natural History 24(3): 595–613. 10.1080/00222939000770401

[B3] Bucklin A Ortman BD Jennings RM Nigro LM Sweetman CJ Copley NJ Sutton T Wiebe PH (2010) A “Rosetta Stone” for metazoan zooplankton: DNA barcode analysis of species diversity of the Sargasso Sea (Northwest Atlantic Ocean). Deep-sea Research. Part II, Topical Studies in Oceanography 57(24–26): 2234–2247. 10.1016/j.dsr2.2010.09.025

[B4] Folmer O Black M Hoeh W Lutz R Vrijenhoek R (1994) DNA primers for amplification of mitochondrial cytochrome c oxidase subunit I from diverse metazoan invertebrates. Molecular Marine Biology and Biotechnology 3: 294–299. 7881515

[B5] Friedlander AM Golbuu Y Caselle JE Ballesteros E Letessier TB Meeuwig JJ Gouezo M Olsudong D Turchik A Sala E (2015) Marine biodiversity and protected areas in Palau: Scientific report to the government of the Republic of Palau.

[B6] Gopalakrishnan G Cornuelle BD (2019) Palau’s effects on regional-scale ocean circulation. Oceanography 32(4): 126–135. 10.5670/oceanog.2019.418

[B7] Hamner WM Colin PL Hamner PP (2007) Export–import dynamics of zooplankton on a coral reef in Palau. Marine Ecology Progress Series 334: 83–92. 10.3354/meps334083

[B8] Ho JS (1980) *Anchicallgusnautili* (willey), a caligid copepod parasitic on nautilus in Palau, with discussion of *Caligulina* Heegaard, 1972. Publications of the Seto Marine Biological Laboratory 25(1–4): 157–165. 10.5134/175987

[B9] Horiguchi T Hayashi Y Kudo H Hara Y (2011) A new benthic dinoflagellate *Spiniferodiniumpalauense* sp. nov. (Dinophyceae) from Palau. Phycologia 50(6): 616–623. 10.2216/11-15.1

[B10] Huys R Gee JM Moore CG Hamond R (1996) Marine and Brackish Water Harpacticoid Copepods. Published for the Linnean Society of London and the Estuarine and Coastal Sciences Association by Field Studies Council, 352 pp.

[B11] Kim IH Boxshall GA (2020) Untold diversity: the astonishing species richness of the Notodelphyidae (Copepoda: Cyclopoida), a family of symbiotic copepods associated with ascidians (Tunicata). Megataxa 4(1): 1–660. 10.11646/megataxa.4.1.1

[B12] Kimura M (1980) A simple method for estimating evolutionary rates of base substitutions through comparative studies of nucleotide sequences. Journal of Molecular Evolution 16(2): 111–120. 10.1007/BF01731581 7463489

[B13] Kumar S Stecher G Tamura K (2016) MEGA7: Molecular evolutionary genetics analysis version 7.0 for bigger datasets. Molecular Biology and Evolution 33(7): 1870–1874. 10.1093/molbev/msw054 27004904 PMC8210823

[B14] Lang K (1944) Monographie der Harpacticiden (Vorläufige Mitteilung). Almqvist & Wiksellls Boktryckeri.

[B15] Lang K (1948) Monographie der Harpacticiden (Vol. 2). Håkan Ohlsson, Lund, 1682 pp.

[B16] Ma L Li XZ (2018) First report of the genus *Rhyncholagena* Lang, 1944 from the South China Sea, with the description of a new species (Crustacea, Copepoda, Harpacticoida, Miraciidae). ZooKeys 805: 15–31. 10.3897/zookeys.805.24331 PMC6299063 30584391

[B17] Marin I Paulay G (2010) *Pinnotherotoniarumphiusi* gen. et sp. nov., a new furry bivalve-associated pontoniine shrimp (Crustacea: Decapoda: Palaemonidae) from Palau. Zootaxa 2636(1): 37–48. 10.11646/zootaxa.2636.1.3

[B18] Myers AA (2013) Amphipoda (Crustacea) from Palau, Micronesia: Families Dexaminidae, Eusiridae, Hyalidae, Ischyroceridae, Leucothoidae and Lysianassidae. Zootaxa 3731(3): 301–323. 10.11646/zootaxa.3731.3.1 25277573

[B19] Myers AA (2014) Amphipoda (Crustacea) from Palau, Micronesia: Families Melphidippidae, Oedicerotidae, Photidae, Pleustidae, Podoceridae, Stenothoidae, Synopiidae and Talitridae. Zootaxa 3811(4): 535–552. 10.11646/zootaxa.3811.4.6 24943185

[B20] Nei M Kumar S (2000) Molecular Evolution and Phylogenetics. Oxford university press, 348 pp.

[B21] Ohtsuka S Kase T Boxshall GA (2000) A new species of *Ridgewayia* (Copepoda: Calanoida) from a submarine cave in Palau, western Pacific. Species Diversity 5(3): 201–213. 10.12782/specdiv.5.201

[B22] Por FD (1964) A study of the Levantine and Pontic Harpacticoida (Crustacea, Copepoda). Zoologische Verhandelinge 64(1): 1–128. 10.1080/00364827.1965.10409556

[B23] Por FD (1967) Level bottom Harpacticoida (Crustacea, Copepoda) from Elat (Red Sea), Part I. Israel Journal of Zoology 16(3): 101–165.

[B24] Saitoh SI Suzuki H Hanzawa N Tamate HB (2011) Species diversity and community structure of pelagic copepods in the marine lakes of Palau. Hydrobiologia 666: 85–97. 10.1007/s10750-010-0095-0

[B25] Sarmento VC Santos PJP (2012) Species of Harpacticoida (Crustacea, Copepoda) from the phytal of Porto de Galinhas coral reefs, northeastern Brazil. Check List 8(5): 936–939. 10.15560/8.5.936

[B26] Thompson JD Higgins DG Gibson TJ (1994) CLUSTAL W: Improving the sensitivity of progressive multiple sequence alignment through sequence weighting, position-specific gap penalties and weight matrix choice. Nucleic Acids Research 22(22): 4673–4680. 10.1093/nar/22.22.4673 7984417 PMC308517

[B27] Wells JBJ (2007) An annotated checklist and keys to the species of CopepodaHarpacticoida (Crustacea). Zootaxa 1568(1): 1–872. 10.11646/zootaxa.1568.1.1

[B28] Williams BD Schrank B Huynh C Shownkeen R Waterston R (1992) A genetic mapping system in *Caenorhabditiselegans* based on polymorphic sequence-tagged sites. Genetics 131(3): 609–624. 10.1093/genetics/131.3.609 1321065 PMC1205034

[B29] Yamaguchi S Endo K (2003) Molecular phylogeny of Ostracoda (Crustacea) inferred from 18S ribosomal DNA sequences: Implication for its origin and diversification. Marine Biology 143(1): 23–38. 10.1007/s00227-003-1062-3

